# Supplementary material for: Comparison of long COVID, recovered COVID, and non-COVID Post-Acute Infection Syndromes over three years
Source: PLoS One. 2025 May 20;20(5):e0323104. doi: 10.1371/journal.pone.0323104 (PMC12092011; doi:10.1371/journal.pone.0323104)
Supplement: S2 Table — (PDF) [file pone.0323104.s002.pdf]

S2 Table. ICD-10 codes used to categorize diagnoses and symptoms in Figs 3-5.

ICD10 Codes	Description	ICD10 Codes	Description	ICD10 Codes	Description
R00	Abnormalities of heart beat	G47.69	Sleep disorders	M25.44	Joint disorder
R00.0	Abnormalities of heart beat	G47.8	Sleep disorders	M25.441	Joint disorder
R00.1	Abnormalities of heart beat	G47.9	Sleep disorders	M25.442	Joint disorder
R00.2	Abnormalities of heart beat	G61	Polyneuropathy and skin sensation disturbances	M25.449	Joint disorder
R00.8	Abnormalities of heart beat	G61.0	Polyneuropathy and skin sensation disturbances	M25.45	Joint disorder
R00.9	Abnormalities of heart beat	G61.1	Polyneuropathy and skin sensation disturbances	M25.451	Joint disorder
I09.0	Pain in throat and chest	G61.8	Polyneuropathy and skin sensation disturbances	M25.452	Joint disorder
I20	Pain in throat and chest	G61.81	Polyneuropathy and skin sensation disturbances	M25.459	Joint disorder
I20.0	Pain in throat and chest	G61.82	Polyneuropathy and skin sensation disturbances	M25.46	Joint disorder
I20.1	Pain in throat and chest	G61.89	Polyneuropathy and skin sensation disturbances	M25.461	Joint disorder
I20.2	Pain in throat and chest	G61.9	Polyneuropathy and skin sensation disturbances	M25.462	Joint disorder
I20.8	Pain in throat and chest	G65	Polyneuropathy and skin sensation disturbances	M25.469	Joint disorder
I20.81	Pain in throat and chest	G65.0	Polyneuropathy and skin sensation disturbances	M25.47	Joint disorder
I20.89	Pain in throat and chest	G65.1	Polyneuropathy and skin sensation disturbances	M25.471	Joint disorder
I20.9	Pain in throat and chest	G65.2	Polyneuropathy and skin sensation disturbances	M25.472	Joint disorder
I21	Myocardial infarction	G89	Unspecified pain	M25.473	Joint disorder
I21.0	Myocardial infarction	G89.0	Unspecified pain	M25.474	Joint disorder
I21.01	Myocardial infarction	G89.1	Unspecified pain	M25.475	Joint disorder
I21.02	Myocardial infarction	G89.11	Unspecified pain	M25.476	Joint disorder
I21.09	Myocardial infarction	G89.12	Unspecified pain	M25.48	Joint disorder
I21.1	Myocardial infarction	G89.18	Unspecified pain	M25.5	Joint disorder
I21.11	Myocardial infarction	G89.2	Unspecified pain	M25.50	Joint disorder

I21.19	Myocardial infarction	G89.21	Unspecified pain	M25.51	Joint disorder
I21.2	Myocardial infarction	G89.22	Unspecified pain	M25.511	Joint disorder
I21.21	Myocardial infarction	G89.28	Unspecified pain	M25.512	Joint disorder
I21.29	Myocardial infarction	G89.29	Unspecified pain	M25.519	Joint disorder
I21.3	Myocardial infarction	G89.3	Unspecified pain	M25.52	Joint disorder
I21.4	Myocardial infarction	G89.4	Unspecified pain	M25.521	Joint disorder
I21.9	Myocardial infarction	F30	Anxiety/Mood disorders	M25.522	Joint disorder
I21.A	Myocardial infarction	F30.1	Anxiety/Mood disorders	M25.529	Joint disorder
I21.A1	Myocardial infarction	F30.10	Anxiety/Mood disorders	M25.53	Joint disorder
I21.A9	Myocardial infarction	F30.11	Anxiety/Mood disorders	M25.531	Joint disorder
I22	Myocardial infarction	F30.12	Anxiety/Mood disorders	M25.532	Joint disorder
I22.0	Myocardial infarction	F30.13	Anxiety/Mood disorders	M25.539	Joint disorder
I22.1	Myocardial infarction	F30.2	Anxiety/Mood disorders	M25.54	Joint disorder
I22.2	Myocardial infarction	F30.3	Anxiety/Mood disorders	M25.541	Joint disorder
I22.8	Myocardial infarction	F30.4	Anxiety/Mood disorders	M25.542	Joint disorder
I22.9	Myocardial infarction	F30.8	Anxiety/Mood disorders	M25.549	Joint disorder
I25.2	Myocardial infarction	F30.9	Anxiety/Mood disorders	M25.55	Joint disorder
I26	Pulmonary embolism	F31	Anxiety/Mood disorders	M25.551	Joint disorder
I26.0	Pulmonary embolism	F31.0	Anxiety/Mood disorders	M25.552	Joint disorder
I26.01	Pulmonary embolism	F31.1	Anxiety/Mood disorders	M25.559	Joint disorder
I26.02	Pulmonary embolism	F31.10	Anxiety/Mood disorders	M25.56	Joint disorder
I26.09	Pulmonary embolism	F31.11	Anxiety/Mood disorders	M25.561	Joint disorder
I26.9	Pulmonary embolism	F31.12	Anxiety/Mood disorders	M25.562	Joint disorder

I26.90	Pulmonary embolism	F31.13	Anxiety/Mood disorders	M25.569	Joint disorder
I26.92	Pulmonary embolism	F31.2	Anxiety/Mood disorders	M25.57	Joint disorder
I26.93	Pulmonary embolism	F31.3	Anxiety/Mood disorders	M25.571	Joint disorder
I26.94	Pulmonary embolism	F31.30	Anxiety/Mood disorders	M25.572	Joint disorder
I26.99	Pulmonary embolism	F31.31	Anxiety/Mood disorders	M25.579	Joint disorder
I40	Pain in throat and chest	F31.32	Anxiety/Mood disorders	M25.59	Joint disorder
I40.0	Pain in throat and chest	F31.4	Anxiety/Mood disorders	M25.6	Joint disorder
I40.1	Pain in throat and chest	F31.5	Anxiety/Mood disorders	M25.60	Joint disorder
I40.8	Pain in throat and chest	F31.6	Anxiety/Mood disorders	M25.61	Joint disorder
I40.9	Pain in throat and chest	F31.60	Anxiety/Mood disorders	M25.611	Joint disorder
I41	Pain in throat and chest	F31.61	Anxiety/Mood disorders	M25.612	Joint disorder
I48	Abnormalities of heart beat	F31.62	Anxiety/Mood disorders	M25.619	Joint disorder
I48.0	Abnormalities of heart beat	F31.63	Anxiety/Mood disorders	M25.62	Joint disorder
I48.1	Abnormalities of heart beat	F31.64	Anxiety/Mood disorders	M25.621	Joint disorder
I48.11	Abnormalities of heart beat	F31.7	Anxiety/Mood disorders	M25.622	Joint disorder
I48.19	Abnormalities of heart beat	F31.70	Anxiety/Mood disorders	M25.629	Joint disorder
I48.2	Abnormalities of heart beat	F31.71	Anxiety/Mood disorders	M25.63	Joint disorder
I48.20	Abnormalities of heart beat	F31.72	Anxiety/Mood disorders	M25.631	Joint disorder
I48.21	Abnormalities of heart beat	F31.73	Anxiety/Mood disorders	M25.632	Joint disorder
I48.3	Abnormalities of heart beat	F31.74	Anxiety/Mood disorders	M25.639	Joint disorder
I48.4	Abnormalities of heart beat	F31.75	Anxiety/Mood disorders	M25.64	Joint disorder
I48.9	Abnormalities of heart beat	F31.76	Anxiety/Mood disorders	M25.641	Joint disorder
I48.91	Abnormalities of heart beat	F31.77	Anxiety/Mood disorders	M25.642	Joint disorder

I48.92	Abnormalities of heart beat	F31.78	Anxiety/Mood disorders	M25.649	Joint disorder
I47	Abnormalities of heart beat	F31.8	Anxiety/Mood disorders	M25.65	Joint disorder
I47.0	Abnormalities of heart beat	F31.81	Anxiety/Mood disorders	M25.651	Joint disorder
I47.1	Abnormalities of heart beat	F31.89	Anxiety/Mood disorders	M25.652	Joint disorder
I47.10	Abnormalities of heart beat	F31.9	Anxiety/Mood disorders	M25.659	Joint disorder
I47.11	Abnormalities of heart beat	F32	Anxiety/Mood disorders	M25.66	Joint disorder
I47.19	Abnormalities of heart beat	F32.0	Anxiety/Mood disorders	M25.661	Joint disorder
I47.2	Abnormalities of heart beat	F32.1	Anxiety/Mood disorders	M25.662	Joint disorder
I47.20	Abnormalities of heart beat	F32.2	Anxiety/Mood disorders	M25.669	Joint disorder
I47.21	Abnormalities of heart beat	F32.3	Anxiety/Mood disorders	M25.67	Joint disorder
I47.29	Abnormalities of heart beat	F32.4	Anxiety/Mood disorders	M25.671	Joint disorder
I47.9	Abnormalities of heart beat	F32.5	Anxiety/Mood disorders	M25.672	Joint disorder
I49	Abnormalities of heart beat	F32.8	Anxiety/Mood disorders	M25.673	Joint disorder
I49.0	Abnormalities of heart beat	F32.81	Anxiety/Mood disorders	M25.674	Joint disorder
I49.01	Abnormalities of heart beat	F32.89	Anxiety/Mood disorders	M25.675	Joint disorder
I49.02	Abnormalities of heart beat	F32.9	Anxiety/Mood disorders	M25.676	Joint disorder
I49.1	Abnormalities of heart beat	F32.A	Anxiety/Mood disorders	M25.69	Joint disorder
I49.2	Abnormalities of heart beat	F33	Anxiety/Mood disorders	M25.7	Joint disorder
I49.3	Abnormalities of heart beat	F33.0	Anxiety/Mood disorders	M25.70	Joint disorder
I49.4	Abnormalities of heart beat	F33.1	Anxiety/Mood disorders	M25.71	Joint disorder
I49.40	Abnormalities of heart beat	F33.2	Anxiety/Mood disorders	M25.711	Joint disorder
I49.49	Abnormalities of heart beat	F33.3	Anxiety/Mood disorders	M25.712	Joint disorder
I49.5	Abnormalities of heart beat	F33.4	Anxiety/Mood disorders	M25.719	Joint disorder

I49.8	Abnormalities of heart beat	F33.40	Anxiety/Mood disorders	M25.72	Joint disorder
I49.9	Abnormalities of heart beat	F33.41	Anxiety/Mood disorders	M25.721	Joint disorder
I51.4	Pain in throat and chest	F33.42	Anxiety/Mood disorders	M25.722	Joint disorder
I60	Hemorrhagic stroke	F33.8	Anxiety/Mood disorders	M25.729	Joint disorder
I60.0	Hemorrhagic stroke	F33.9	Anxiety/Mood disorders	M25.73	Joint disorder
I60.00	Hemorrhagic stroke	F34	Anxiety/Mood disorders	M25.731	Joint disorder
I60.01	Hemorrhagic stroke	F34.0	Anxiety/Mood disorders	M25.732	Joint disorder
I60.02	Hemorrhagic stroke	F34.1	Anxiety/Mood disorders	M25.739	Joint disorder
I60.1	Hemorrhagic stroke	F34.8	Anxiety/Mood disorders	M25.74	Joint disorder
I60.10	Hemorrhagic stroke	F34.81	Anxiety/Mood disorders	M25.741	Joint disorder
I60.11	Hemorrhagic stroke	F34.89	Anxiety/Mood disorders	M25.742	Joint disorder
I60.12	Hemorrhagic stroke	F34.9	Anxiety/Mood disorders	M25.749	Joint disorder
I60.2	Hemorrhagic stroke	F39	Anxiety/Mood disorders	M25.75	Joint disorder
I60.3	Hemorrhagic stroke	F40	Anxiety/Mood disorders	M25.751	Joint disorder
I60.30	Hemorrhagic stroke	F40.00	Anxiety/Mood disorders	M25.752	Joint disorder
I60.31	Hemorrhagic stroke	F40.000	Anxiety/Mood disorders	M25.759	Joint disorder
I60.32	Hemorrhagic stroke	F40.010	Anxiety/Mood disorders	M25.76	Joint disorder
I60.4	Hemorrhagic stroke	F40.020	Anxiety/Mood disorders	M25.761	Joint disorder
I60.5	Hemorrhagic stroke	F40.10	Anxiety/Mood disorders	M25.762	Joint disorder
I60.50	Hemorrhagic stroke	F40.100	Anxiety/Mood disorders	M25.769	Joint disorder
I60.51	Hemorrhagic stroke	F40.110	Anxiety/Mood disorders	M25.77	Joint disorder
I60.52	Hemorrhagic stroke	F40.2	Anxiety/Mood disorders	M25.771	Joint disorder
I60.6	Hemorrhagic stroke	F40.21	Anxiety/Mood disorders	M25.772	Joint disorder

I60.7	Hemorrhagic stroke	F40.210	Anxiety/Mood disorders	M25.773	Joint disorder
I60.8	Hemorrhagic stroke	F40.218	Anxiety/Mood disorders	M25.774	Joint disorder
I60.9	Hemorrhagic stroke	F40.22	Anxiety/Mood disorders	M25.775	Joint disorder
I61	Hemorrhagic stroke	F40.220	Anxiety/Mood disorders	M25.776	Joint disorder
I61.0	Hemorrhagic stroke	F40.228	Anxiety/Mood disorders	M25.78	Joint disorder
I61.1	Hemorrhagic stroke	F40.23	Anxiety/Mood disorders	M25.8	Joint disorder
I61.2	Hemorrhagic stroke	F40.230	Anxiety/Mood disorders	M25.80	Joint disorder
I61.3	Hemorrhagic stroke	F40.231	Anxiety/Mood disorders	M25.81	Joint disorder
I61.4	Hemorrhagic stroke	F40.232	Anxiety/Mood disorders	M25.811	Joint disorder
I61.5	Hemorrhagic stroke	F40.233	Anxiety/Mood disorders	M25.812	Joint disorder
I61.6	Hemorrhagic stroke	F40.24	Anxiety/Mood disorders	M25.819	Joint disorder
I61.8	Hemorrhagic stroke	F40.240	Anxiety/Mood disorders	M25.82	Joint disorder
I61.9	Hemorrhagic stroke	F40.241	Anxiety/Mood disorders	M25.821	Joint disorder
I63	Ischemic stroke	F40.242	Anxiety/Mood disorders	M25.822	Joint disorder
I63.0	Ischemic stroke	F40.243	Anxiety/Mood disorders	M25.829	Joint disorder
I63.00	Ischemic stroke	F40.248	Anxiety/Mood disorders	M25.83	Joint disorder
I63.01	Ischemic stroke	F40.29	Anxiety/Mood disorders	M25.831	Joint disorder
I63.011	Ischemic stroke	F40.290	Anxiety/Mood disorders	M25.832	Joint disorder
I63.012	Ischemic stroke	F40.291	Anxiety/Mood disorders	M25.839	Joint disorder
I63.013	Ischemic stroke	F40.298	Anxiety/Mood disorders	M25.84	Joint disorder
I63.019	Ischemic stroke	F40.8	Anxiety/Mood disorders	M25.841	Joint disorder
I63.02	Ischemic stroke	F40.9	Anxiety/Mood disorders	M25.842	Joint disorder
I63.03	Ischemic stroke	F41	Anxiety/Mood disorders	M25.849	Joint disorder

I63.031	Ischemic stroke	F41.0	Anxiety/Mood disorders	M25.85	Joint disorder
I63.032	Ischemic stroke	F41.1	Anxiety/Mood disorders	M25.851	Joint disorder
I63.033	Ischemic stroke	F41.3	Anxiety/Mood disorders	M25.852	Joint disorder
I63.039	Ischemic stroke	F41.8	Anxiety/Mood disorders	M25.859	Joint disorder
I63.09	Ischemic stroke	F41.9	Anxiety/Mood disorders	M25.86	Joint disorder
I63.1	Ischemic stroke	R11	Nausea and vomiting	M25.861	Joint disorder
I63.10	Ischemic stroke	R11.0	Nausea and vomiting	M25.862	Joint disorder
I63.11	Ischemic stroke	R11.1	Nausea and vomiting	M25.869	Joint disorder
I63.111	Ischemic stroke	R11.10	Nausea and vomiting	M25.87	Joint disorder
I63.112	Ischemic stroke	R11.11	Nausea and vomiting	M25.871	Joint disorder
I63.113	Ischemic stroke	R11.12	Nausea and vomiting	M25.872	Joint disorder
I63.119	Ischemic stroke	R11.13	Nausea and vomiting	M25.879	Joint disorder
I63.12	Ischemic stroke	R11.14	Nausea and vomiting	M25.9	Joint disorder
I63.13	Ischemic stroke	R11.15	Nausea and vomiting	M79	soft tissue disorders
I63.131	Ischemic stroke	R11.2	Nausea and vomiting	M79.0	soft tissue disorders
I63.132	Ischemic stroke	R20	Polyneuropathy and skin sensation disturbances	M79.1	soft tissue disorders
I63.133	Ischemic stroke	R20.0	Polyneuropathy and skin sensation disturbances	M79.10	soft tissue disorders
I63.139	Ischemic stroke	R20.1	Polyneuropathy and skin sensation disturbances	M79.11	soft tissue disorders
I63.19	Ischemic stroke	R20.2	Polyneuropathy and skin sensation disturbances	M79.12	soft tissue disorders
I63.2	Ischemic stroke	R20.3	Polyneuropathy and skin sensation disturbances	M79.18	soft tissue disorders
I63.20	Ischemic stroke	R20.8	Polyneuropathy and skin sensation disturbances	M79.2	soft tissue disorders
I63.21	Ischemic stroke	R20.9	Polyneuropathy and skin sensation disturbances	M79.3	soft tissue disorders
I63.211	Ischemic stroke	R41	Cognition, perception, or emotional state symptoms	M79.4	soft tissue disorders

I63.212	Ischemic stroke	R41.0	Cognition, perception, or emotional state symptoms	M79.5	soft tissue disorders
I63.213	Ischemic stroke	R41.1	Cognition, perception, or emotional state symptoms	M79.6	soft tissue disorders
I63.219	Ischemic stroke	R41.2	Cognition, perception, or emotional state symptoms	M79.60	soft tissue disorders
I63.22	Ischemic stroke	R41.3	Cognition, perception, or emotional state symptoms	M79.601	soft tissue disorders
I63.23	Ischemic stroke	R41.4	Cognition, perception, or emotional state symptoms	M79.602	soft tissue disorders
I63.231	Ischemic stroke	R41.8	Cognition, perception, or emotional state symptoms	M79.603	soft tissue disorders
I63.232	Ischemic stroke	R41.81	Cognition, perception, or emotional state symptoms	M79.604	soft tissue disorders
I63.233	Ischemic stroke	R41.82	Cognition, perception, or emotional state symptoms	M79.605	soft tissue disorders
I63.239	Ischemic stroke	R41.83	Cognition, perception, or emotional state symptoms	M79.606	soft tissue disorders
I63.29	Ischemic stroke	R41.84	Cognition, perception, or emotional state symptoms	M79.609	soft tissue disorders
I63.3	Ischemic stroke	R41.840	Cognition, perception, or emotional state symptoms	M79.62	soft tissue disorders
I63.30	Ischemic stroke	R41.841	Cognition, perception, or emotional state symptoms	M79.621	soft tissue disorders
I63.31	Ischemic stroke	R41.842	Cognition, perception, or emotional state symptoms	M79.622	soft tissue disorders
I63.311	Ischemic stroke	R41.843	Cognition, perception, or emotional state symptoms	M79.629	soft tissue disorders
I63.312	Ischemic stroke	R41.844	Cognition, perception, or emotional state symptoms	M79.63	soft tissue disorders
I63.313	Ischemic stroke	R41.89	Cognition, perception, or emotional state symptoms	M79.631	soft tissue disorders
I63.319	Ischemic stroke	R41.9	Cognition, perception, or emotional state symptoms	M79.632	soft tissue disorders
I63.32	Ischemic stroke	I10	Hypertension	M79.639	soft tissue disorders
I63.321	Ischemic stroke	I16	Hypertension	M79.64	soft tissue disorders
I63.322	Ischemic stroke	I16.0	Hypertension	M79.641	soft tissue disorders
I63.323	Ischemic stroke	I16.1	Hypertension	M79.642	soft tissue disorders
I63.329	Ischemic stroke	I16.9	Hypertension	M79.643	soft tissue disorders
I63.33	Ischemic stroke	E03	Hypothyroidism	M79.644	soft tissue disorders

I63.331	Ischemic stroke	E03.0	Hypothyroidism	M79.645	soft tissue disorders
I63.332	Ischemic stroke	E03.1	Hypothyroidism	M79.646	soft tissue disorders
I63.333	Ischemic stroke	E03.2	Hypothyroidism	M79.65	soft tissue disorders
I63.339	Ischemic stroke	E03.3	Hypothyroidism	M79.651	soft tissue disorders
I63.34	Ischemic stroke	E03.4	Hypothyroidism	M79.652	soft tissue disorders
I63.341	Ischemic stroke	E03.5	Hypothyroidism	M79.659	soft tissue disorders
I63.342	Ischemic stroke	E03.8	Hypothyroidism	M79.66	soft tissue disorders
I63.343	Ischemic stroke	E03.9	Hypothyroidism	M79.661	soft tissue disorders
I63.349	Ischemic stroke	E10.2	Diabetes mellitus	M79.662	soft tissue disorders
I63.39	Ischemic stroke	E10.21	Diabetes mellitus	M79.669	soft tissue disorders
I63.4	Ischemic stroke	E10.22	Diabetes mellitus	M79.67	soft tissue disorders
I63.40	Ischemic stroke	E10.29	Diabetes mellitus	M79.671	soft tissue disorders
I63.41	Ischemic stroke	E10.3	Diabetes mellitus	M79.672	soft tissue disorders
I63.411	Ischemic stroke	E10.31	Diabetes mellitus	M79.673	soft tissue disorders
I63.412	Ischemic stroke	E10.311	Diabetes mellitus	M79.674	soft tissue disorders
I63.413	Ischemic stroke	E10.319	Diabetes mellitus	M79.675	soft tissue disorders
I63.419	Ischemic stroke	E10.32	Diabetes mellitus	M79.676	soft tissue disorders
I63.42	Ischemic stroke	E10.321	Diabetes mellitus	M79.7	soft tissue disorders
I63.421	Ischemic stroke	E10.329	Diabetes mellitus	M79.8	soft tissue disorders
I63.422	Ischemic stroke	E10.33	Diabetes mellitus	M79.81	soft tissue disorders
I63.423	Ischemic stroke	E10.331	Diabetes mellitus	M79.89	soft tissue disorders
I63.429	Ischemic stroke	E10.339	Diabetes mellitus	M79.9	soft tissue disorders
I63.43	Ischemic stroke	E10.34	Diabetes mellitus	M79.A	soft tissue disorders

I63.431	Ischemic stroke	E10.341	Diabetes mellitus	M79.A1	soft tissue disorders
I63.432	Ischemic stroke	E10.349	Diabetes mellitus	M79.A11	soft tissue disorders
I63.433	Ischemic stroke	E10.35	Diabetes mellitus	M79.A12	soft tissue disorders
I63.439	Ischemic stroke	E10.351	Diabetes mellitus	M79.A19	soft tissue disorders
I63.44	Ischemic stroke	E10.352	Diabetes mellitus	M79.A2	soft tissue disorders
I63.441	Ischemic stroke	E10.353	Diabetes mellitus	M79.A21	soft tissue disorders
I63.442	Ischemic stroke	E10.354	Diabetes mellitus	M79.A22	soft tissue disorders
I63.443	Ischemic stroke	E10.355	Diabetes mellitus	M79.A29	soft tissue disorders
I63.449	Ischemic stroke	E10.359	Diabetes mellitus	M79.A3	soft tissue disorders
I63.49	Ischemic stroke	E10.36	Diabetes mellitus	M79.A9	soft tissue disorders
I63.5	Ischemic stroke	E10.37	Diabetes mellitus	M54	Dorsalgia
I63.50	Ischemic stroke	E10.39	Diabetes mellitus	M54.0	Dorsalgia
I63.51	Ischemic stroke	E10.4	Diabetes mellitus	M54.00	Dorsalgia
I63.511	Ischemic stroke	E10.40	Diabetes mellitus	M54.01	Dorsalgia
I63.512	Ischemic stroke	E10.41	Diabetes mellitus	M54.02	Dorsalgia
I63.513	Ischemic stroke	E10.42	Diabetes mellitus	M54.03	Dorsalgia
I63.519	Ischemic stroke	E10.43	Diabetes mellitus	M54.04	Dorsalgia
I63.52	Ischemic stroke	E10.44	Diabetes mellitus	M54.05	Dorsalgia
I63.521	Ischemic stroke	E10.49	Diabetes mellitus	M54.06	Dorsalgia
I63.522	Ischemic stroke	E10.5	Diabetes mellitus	M54.07	Dorsalgia
I63.523	Ischemic stroke	E10.51	Diabetes mellitus	M54.08	Dorsalgia
I63.529	Ischemic stroke	E10.52	Diabetes mellitus	M54.09	Dorsalgia
I63.53	Ischemic stroke	E10.59	Diabetes mellitus	M54.1	Dorsalgia
I63.531	Ischemic stroke	E11	Diabetes mellitus	M54.10	Dorsalgia
I63.532	Ischemic stroke	E11.0	Diabetes mellitus	M54.11	Dorsalgia
I63.533	Ischemic stroke	E11.00	Diabetes mellitus	M54.12	Dorsalgia
I63.539	Ischemic stroke	E11.01	Diabetes mellitus	M54.13	Dorsalgia
I63.54	Ischemic stroke	E11.1	Diabetes mellitus	M54.14	Dorsalgia
I63.541	Ischemic stroke	E11.10	Diabetes mellitus	M54.15	Dorsalgia
I63.542	Ischemic stroke	E11.11	Diabetes mellitus	M54.16	Dorsalgia
I63.543	Ischemic stroke	E11.6	Diabetes mellitus	M54.17	Dorsalgia
I63.549	Ischemic stroke	E11.61	Diabetes mellitus	M54.18	Dorsalgia
I63.59	Ischemic stroke	E11.610	Diabetes mellitus	M54.2	Dorsalgia
I63.6	Ischemic stroke	E11.618	Diabetes mellitus	M54.3	Dorsalgia

I63.8	Ischemic stroke	E11.62	Diabetes mellitus	M54.30	Dorsalgia
I63.81	Ischemic stroke	E11.620	Diabetes mellitus	M54.31	Dorsalgia
I63.89	Ischemic stroke	E11.621	Diabetes mellitus	M54.32	Dorsalgia
I63.9	Ischemic stroke	E11.622	Diabetes mellitus	M54.4	Dorsalgia
I65	Ischemic stroke	E11.628	Diabetes mellitus	M54.40	Dorsalgia
I65.0	Ischemic stroke	E11.63	Diabetes mellitus	M54.41	Dorsalgia
I65.01	Ischemic stroke	E11.630	Diabetes mellitus	M54.42	Dorsalgia
I65.02	Ischemic stroke	E11.638	Diabetes mellitus	M54.5	Dorsalgia
I65.03	Ischemic stroke	E11.64	Diabetes mellitus	M54.50	Dorsalgia
I65.09	Ischemic stroke	E11.641	Diabetes mellitus	M54.51	Dorsalgia
I65.1	Ischemic stroke	E11.649	Diabetes mellitus	M54.59	Dorsalgia
I65.2	Ischemic stroke	E11.65	Diabetes mellitus	M54.6	Dorsalgia
I65.21	Ischemic stroke	E11.69	Diabetes mellitus	M54.8	Dorsalgia
I65.22	Ischemic stroke	E11.8	Diabetes mellitus	M54.81	Dorsalgia
I65.23	Ischemic stroke	E11.9	Diabetes mellitus	M54.89	Dorsalgia
I65.29	Ischemic stroke	E66	Overweight and obesity	M54.9	Dorsalgia
I65.8	Ischemic stroke	E66.0	Overweight and obesity	M60	Muscle disorders
I65.9	Ischemic stroke	E66.01	Overweight and obesity	M60.0	Muscle disorders
I66	Ischemic stroke	E66.09	Overweight and obesity	M60.00	Muscle disorders
I66.0	Ischemic stroke	E66.1	Overweight and obesity	M60.000	Muscle disorders
I66.01	Ischemic stroke	E66.2	Overweight and obesity	M60.001	Muscle disorders
I66.02	Ischemic stroke	E66.3	Overweight and obesity	M60.002	Muscle disorders
I66.03	Ischemic stroke	E66.8	Overweight and obesity	M60.003	Muscle disorders
I66.09	Ischemic stroke	E66.9	Overweight and obesity	M60.004	Muscle disorders
I66.1	Ischemic stroke	Z68.3	Overweight and obesity	M60.005	Muscle disorders
I66.11	Ischemic stroke	Z68.30	Overweight and obesity	M60.009	Muscle disorders
I66.12	Ischemic stroke	Z68.31	Overweight and obesity	M60.01	Muscle disorders
I66.13	Ischemic stroke	Z68.32	Overweight and obesity	M60.011	Muscle disorders
I66.19	Ischemic stroke	Z68.33	Overweight and obesity	M60.012	Muscle disorders
I66.2	Ischemic stroke	Z68.34	Overweight and obesity	M60.019	Muscle disorders

I66.21	Ischemic stroke	Z68.35	Overweight and obesity	M60.02	Muscle disorders
I66.22	Ischemic stroke	Z68.36	Overweight and obesity	M60.021	Muscle disorders
I66.23	Ischemic stroke	Z68.37	Overweight and obesity	M60.022	Muscle disorders
I66.29	Ischemic stroke	Z68.38	Overweight and obesity	M60.029	Muscle disorders
I66.3	Ischemic stroke	Z68.39	Overweight and obesity	M60.03	Muscle disorders
I66.8	Ischemic stroke	Z68.4	Overweight and obesity	M60.031	Muscle disorders
I66.9	Ischemic stroke	Z68.41	Overweight and obesity	M60.032	Muscle disorders
R05	Cough	Z68.42	Overweight and obesity	M60.039	Muscle disorders
R05.1	Cough	Z68.43	Overweight and obesity	M60.04	Muscle disorders
R05.2	Cough	Z68.44	Overweight and obesity	M60.041	Muscle disorders
R05.3	Cough	Z68.45	Overweight and obesity	M60.042	Muscle disorders
R05.4	Cough	E78	Disorders of lipoprotein metabolism	M60.043	Muscle disorders
R05.8	Cough	E78.0	Disorders of lipoprotein metabolism	M60.044	Muscle disorders
R05.9	Cough	E78.00	Disorders of lipoprotein metabolism	M60.045	Muscle disorders
R06	Abnormalities of breathing	E78.01	Disorders of lipoprotein metabolism	M60.046	Muscle disorders
R06.0	Abnormalities of breathing	E78.1	Disorders of lipoprotein metabolism	M60.05	Muscle disorders
R06.00	Abnormalities of breathing	E78.2	Disorders of lipoprotein metabolism	M60.051	Muscle disorders
R06.01	Abnormalities of breathing	E78.3	Disorders of lipoprotein metabolism	M60.052	Muscle disorders
R06.02	Abnormalities of breathing	E78.4	Disorders of lipoprotein metabolism	M60.059	Muscle disorders
R06.03	Abnormalities of breathing	E78.41	Disorders of lipoprotein metabolism	M60.06	Muscle disorders
R06.09	Abnormalities of breathing	E78.49	Disorders of lipoprotein metabolism	M60.061	Muscle disorders
R06.1	Abnormalities of breathing	E78.5	Disorders of lipoprotein metabolism	M60.062	Muscle disorders
R06.2	Abnormalities of breathing	E78.6	Disorders of lipoprotein metabolism	M60.069	Muscle disorders

R06.3	Abnormalities of breathing	E78.7	Disorders of lipoprotein metabolism	M60.07	Muscle disorders
R06.4	Abnormalities of breathing	E78.70	Disorders of lipoprotein metabolism	M60.070	Muscle disorders
R06.5	Abnormalities of breathing	E78.71	Disorders of lipoprotein metabolism	M60.071	Muscle disorders
R06.6	Abnormalities of breathing	E78.72	Disorders of lipoprotein metabolism	M60.072	Muscle disorders
R06.7	Abnormalities of breathing	E78.79	Disorders of lipoprotein metabolism	M60.073	Muscle disorders
R06.8	Abnormalities of breathing	E78.8	Disorders of lipoprotein metabolism	M60.074	Muscle disorders
R06.81	Abnormalities of breathing	E78.81	Disorders of lipoprotein metabolism	M60.075	Muscle disorders
R06.82	Abnormalities of breathing	E78.89	Disorders of lipoprotein metabolism	M60.076	Muscle disorders
R06.83	Abnormalities of breathing	E78.9	Disorders of lipoprotein metabolism	M60.077	Muscle disorders
R06.89	Abnormalities of breathing	E83	Disorders of mineral metabolism	M60.078	Muscle disorders
R06.9	Abnormalities of breathing	E83.0	Disorders of mineral metabolism	M60.08	Muscle disorders
R07	Pain in throat and chest	E83.00	Disorders of mineral metabolism	M60.09	Muscle disorders
R07.0	Pain in throat and chest	E83.01	Disorders of mineral metabolism	M60.1	Muscle disorders
R07.1	Pain in throat and chest	E83.09	Disorders of mineral metabolism	M60.10	Muscle disorders
R07.2	Pain in throat and chest	E83.1	Disorders of mineral metabolism	M60.11	Muscle disorders
R07.8	Pain in throat and chest	E83.10	Disorders of mineral metabolism	M60.111	Muscle disorders
R07.81	Pain in throat and chest	E83.11	Disorders of mineral metabolism	M60.112	Muscle disorders
R07.82	Pain in throat and chest	E83.11	Disorders of mineral metabolism	M60.119	Muscle disorders
R07.89	Pain in throat and chest	E83.11	Disorders of mineral metabolism	M60.12	Muscle disorders
R07.9	Pain in throat and chest	E83.11	Disorders of mineral metabolism	M60.121	Muscle disorders
R09	Other circulatory and respiratory symptoms	E83.11	Disorders of mineral metabolism	M60.122	Muscle disorders
R09.0	Other circulatory and respiratory symptoms	E83.19	Disorders of mineral metabolism	M60.129	Muscle disorders
R09.01	Other circulatory and respiratory symptoms	E83.2	Disorders of mineral metabolism	M60.13	Muscle disorders

R09.02	Other circulatory and respiratory symptoms	E83.3	Disorders of mineral metabolism	M60.131	Muscle disorders
R09.1	Other circulatory and respiratory symptoms	E83.30	Disorders of mineral metabolism	M60.132	Muscle disorders
R09.2	Other circulatory and respiratory symptoms	E83.31	Disorders of mineral metabolism	M60.139	Muscle disorders
R09.3	Other circulatory and respiratory symptoms	E83.32	Disorders of mineral metabolism	M60.14	Muscle disorders
R09.8	Other circulatory and respiratory symptoms	E83.39	Disorders of mineral metabolism	M60.141	Muscle disorders
R09.81	Other circulatory and respiratory symptoms	E83.4	Disorders of mineral metabolism	M60.142	Muscle disorders
R09.82	Other circulatory and respiratory symptoms	E83.40	Disorders of mineral metabolism	M60.149	Muscle disorders
R09.89	Other circulatory and respiratory symptoms	E83.41	Disorders of mineral metabolism	M60.15	Muscle disorders
R09.A	Other circulatory and respiratory symptoms	E83.42	Disorders of mineral metabolism	M60.151	Muscle disorders
R09.A0	Other circulatory and respiratory symptoms	E83.49	Disorders of mineral metabolism	M60.152	Muscle disorders
R09.A1	Other circulatory and respiratory symptoms	E83.5	Disorders of mineral metabolism	M60.159	Muscle disorders
R09.A2	Other circulatory and respiratory symptoms	E83.50	Disorders of mineral metabolism	M60.16	Muscle disorders
R09.A9	Other circulatory and respiratory symptoms	E83.51	Disorders of mineral metabolism	M60.161	Muscle disorders
R65	Systemic inflammation	E83.52	Disorders of mineral metabolism	M60.162	Muscle disorders
R65.1	Systemic inflammation	E83.59	Disorders of mineral metabolism	M60.169	Muscle disorders
R65.10	Systemic inflammation	E83.8	Disorders of mineral metabolism	M60.17	Muscle disorders
R65.11	Systemic inflammation	E83.81	Disorders of mineral metabolism	M60.171	Muscle disorders
R65.2	Systemic inflammation	E83.89	Disorders of mineral metabolism	M60.172	Muscle disorders
R65.20	Systemic inflammation	E83.9	Disorders of mineral metabolism	M60.179	Muscle disorders
R65.21	Systemic inflammation	E87	Fluid, electrolyte and acid-base disorders	M60.18	Muscle disorders
R91	Abnormal findings on diagnostic imaging of lung	E87.0	Fluid, electrolyte and acid-base disorders	M60.19	Muscle disorders
R91.1	Abnormal findings on diagnostic imaging of lung	E87.1	Fluid, electrolyte and acid-base disorders	M60.2	Muscle disorders
R91.8	Abnormal findings on diagnostic imaging of lung	E87.2	Fluid, electrolyte and acid-base disorders	M60.20	Muscle disorders

J06	Acute upper respiratory infections	E87.20	Fluid, electrolyte and acid-base disorders	M60.21	Muscle disorders
J06.0	Acute upper respiratory infections	E87.21	Fluid, electrolyte and acid-base disorders	M60.211	Muscle disorders
J06.9	Acute upper respiratory infections	E87.22	Fluid, electrolyte and acid-base disorders	M60.212	Muscle disorders
J12	Pneumonia	E87.29	Fluid, electrolyte and acid-base disorders	M60.219	Muscle disorders
J12.0	Pneumonia	E87.3	Fluid, electrolyte and acid-base disorders	M60.22	Muscle disorders
J12.1	Pneumonia	E87.4	Fluid, electrolyte and acid-base disorders	M60.221	Muscle disorders
J12.2	Pneumonia	E87.5	Fluid, electrolyte and acid-base disorders	M60.222	Muscle disorders
J12.3	Pneumonia	E87.6	Fluid, electrolyte and acid-base disorders	M60.229	Muscle disorders
J12.8	Pneumonia	E87.7	Fluid, electrolyte and acid-base disorders	M60.23	Muscle disorders
J12.81	Pneumonia	E87.70	Fluid, electrolyte and acid-base disorders	M60.231	Muscle disorders
J12.82	Pneumonia	E87.71	Fluid, electrolyte and acid-base disorders	M60.232	Muscle disorders
J12.89	Pneumonia	E87.79	Fluid, electrolyte and acid-base disorders	M60.239	Muscle disorders
J12.9	Pneumonia	E87.8	Fluid, electrolyte and acid-base disorders	M60.24	Muscle disorders
J18	Pneumonia	R10	Digestive or abdominal symptoms	M60.241	Muscle disorders
J18.0	Pneumonia	R10.0	Digestive or abdominal symptoms	M60.242	Muscle disorders
J18.1	Pneumonia	R10.1	Digestive or abdominal symptoms	M60.249	Muscle disorders
J18.2	Pneumonia	R10.10	Digestive or abdominal symptoms	M60.25	Muscle disorders
J18.8	Pneumonia	R10.11	Digestive or abdominal symptoms	M60.251	Muscle disorders
J18.9	Pneumonia	R10.12	Digestive or abdominal symptoms	M60.252	Muscle disorders
J22	Acute lower respiratory infection	R10.13	Digestive or abdominal symptoms	M60.259	Muscle disorders
J45	Asthma	R10.2	Digestive or abdominal symptoms	M60.26	Muscle disorders
J45.2	Asthma	R10.3	Digestive or abdominal symptoms	M60.261	Muscle disorders
J45.20	Asthma	R10.30	Digestive or abdominal symptoms	M60.262	Muscle disorders

J45.21	Asthma	R10.31	Digestive or abdominal symptoms	M60.269	Muscle disorders
J45.22	Asthma	R10.32	Digestive or abdominal symptoms	M60.27	Muscle disorders
J45.3	Asthma	R10.33	Digestive or abdominal symptoms	M60.271	Muscle disorders
J45.30	Asthma	R10.8	Digestive or abdominal symptoms	M60.272	Muscle disorders
J45.31	Asthma	R10.81	Digestive or abdominal symptoms	M60.279	Muscle disorders
J45.32	Asthma	R10.81	Digestive or abdominal symptoms	M60.28	Muscle disorders
J45.4	Asthma	R10.81	Digestive or abdominal symptoms	M60.8	Muscle disorders
J45.40	Asthma	R10.81	Digestive or abdominal symptoms	M60.80	Muscle disorders
J45.41	Asthma	R10.81	Digestive or abdominal symptoms	M60.81	Muscle disorders
J45.42	Asthma	R10.81	Digestive or abdominal symptoms	M60.811	Muscle disorders
J45.5	Asthma	R10.81	Digestive or abdominal symptoms	M60.812	Muscle disorders
J45.50	Asthma	R10.81	Digestive or abdominal symptoms	M60.819	Muscle disorders
J45.51	Asthma	R10.81	Digestive or abdominal symptoms	M60.82	Muscle disorders
J45.52	Asthma	R10.82	Digestive or abdominal symptoms	M60.821	Muscle disorders
J45.9	Asthma	R10.82	Digestive or abdominal symptoms	M60.822	Muscle disorders
J45.90	Asthma	R10.82	Digestive or abdominal symptoms	M60.829	Muscle disorders
J45.901	Asthma	R10.82	Digestive or abdominal symptoms	M60.83	Muscle disorders
J45.902	Asthma	R10.82	Digestive or abdominal symptoms	M60.831	Muscle disorders
J45.909	Asthma	R10.82	Digestive or abdominal symptoms	M60.832	Muscle disorders
J45.99	Asthma	R10.82	Digestive or abdominal symptoms	M60.839	Muscle disorders
J45.990	Asthma	R10.82	Digestive or abdominal symptoms	M60.84	Muscle disorders
J45.991	Asthma	R10.82	Digestive or abdominal symptoms	M60.841	Muscle disorders
J45.998	Asthma	R10.83	Digestive or abdominal symptoms	M60.842	Muscle disorders

J44	COPD or Interstitial lung disease	R10.84	Digestive or abdominal symptoms	M60.849	Muscle disorders
J44.0	COPD or Interstitial lung disease	R10.9	Digestive or abdominal symptoms	M60.85	Muscle disorders
J44.1	COPD or Interstitial lung disease	R19	Digestive or abdominal symptoms	M60.851	Muscle disorders
J44.8	Other chronic obstructive pulmonary disease	R19.0	Digestive or abdominal symptoms	M60.852	Muscle disorders
J44.81	COPD or Interstitial lung disease	R19.00	Digestive or abdominal symptoms	M60.859	Muscle disorders
J44.89	COPD or Interstitial lung disease	R19.01	Digestive or abdominal symptoms	M60.86	Muscle disorders
J44.9	COPD or Interstitial lung disease	R19.02	Digestive or abdominal symptoms	M60.861	Muscle disorders
J80	COPD or Interstitial lung disease	R19.03	Digestive or abdominal symptoms	M60.862	Muscle disorders
J84	COPD or Interstitial lung disease	R19.04	Digestive or abdominal symptoms	M60.869	Muscle disorders
J84.0	COPD or Interstitial lung disease	R19.05	Digestive or abdominal symptoms	M60.87	Muscle disorders
J84.01	COPD or Interstitial lung disease	R19.06	Digestive or abdominal symptoms	M60.871	Muscle disorders
J84.02	COPD or Interstitial lung disease	R19.07	Digestive or abdominal symptoms	M60.872	Muscle disorders
J84.03	COPD or Interstitial lung disease	R19.09	Digestive or abdominal symptoms	M60.879	Muscle disorders
J84.09	COPD or Interstitial lung disease	R19.1	Digestive or abdominal symptoms	M60.88	Muscle disorders
J84.1	COPD or Interstitial lung disease	R19.11	Digestive or abdominal symptoms	M60.89	Muscle disorders
J84.10	COPD or Interstitial lung disease	R19.12	Digestive or abdominal symptoms	M60.9	Muscle disorders
J84.11	COPD or Interstitial lung disease	R19.15	Digestive or abdominal symptoms	M62	Muscle disorders
J84.111	COPD or Interstitial lung disease	R19.2	Digestive or abdominal symptoms	M62.0	Muscle disorders
J84.112	COPD or Interstitial lung disease	R19.3	Digestive or abdominal symptoms	M62.00	Muscle disorders
J84.113	COPD or Interstitial lung disease	R19.30	Digestive or abdominal symptoms	M62.01	Muscle disorders
J84.114	COPD or Interstitial lung disease	R19.31	Digestive or abdominal symptoms	M62.011	Muscle disorders
J84.115	COPD or Interstitial lung disease	R19.32	Digestive or abdominal symptoms	M62.012	Muscle disorders
J84.116	COPD or Interstitial lung disease	R19.33	Digestive or abdominal symptoms	M62.019	Muscle disorders

J84.117	COPD or Interstitial lung disease	R19.34	Digestive or abdominal symptoms	M62.02	Muscle disorders
J84.17	COPD or Interstitial lung disease	R19.35	Digestive or abdominal symptoms	M62.021	Muscle disorders
J84.170	COPD or Interstitial lung disease	R19.36	Digestive or abdominal symptoms	M62.022	Muscle disorders
J84.178	COPD or Interstitial lung disease	R19.37	Digestive or abdominal symptoms	M62.029	Muscle disorders
J84.2	COPD or Interstitial lung disease	R19.4	Digestive or abdominal symptoms	M62.03	Muscle disorders
J84.8	COPD or Interstitial lung disease	R19.5	Digestive or abdominal symptoms	M62.031	Muscle disorders
J84.81	COPD or Interstitial lung disease	R19.6	Digestive or abdominal symptoms	M62.032	Muscle disorders
J84.82	COPD or Interstitial lung disease	R19.7	Digestive or abdominal symptoms	M62.039	Muscle disorders
J84.83	COPD or Interstitial lung disease	R19.8	Digestive or abdominal symptoms	M62.04	Muscle disorders
J84.84	COPD or Interstitial lung disease	K21	Gastro-esophageal reflux disease	M62.041	Muscle disorders
J84.841	COPD or Interstitial lung disease	K21.0	Gastro-esophageal reflux disease	M62.042	Muscle disorders
J84.842	COPD or Interstitial lung disease	K21.0	Gastro-esophageal reflux disease	M62.049	Muscle disorders
J84.843	COPD or Interstitial lung disease	K21.0	Gastro-esophageal reflux disease	M62.05	Muscle disorders
J84.848	COPD or Interstitial lung disease	K21.9	Gastro-esophageal reflux disease	M62.051	Muscle disorders
J84.89	COPD or Interstitial lung disease	K59	Functional intestinal disorders	M62.052	Muscle disorders
J84.9	COPD or Interstitial lung disease	K59.0	Functional intestinal disorders	M62.059	Muscle disorders
J90	Other respiratory disorders	K59.00	Functional intestinal disorders	M62.06	Muscle disorders
J96	Respiratory failure	K59.01	Functional intestinal disorders	M62.061	Muscle disorders
J96.0	Respiratory failure	K59.02	Functional intestinal disorders	M62.062	Muscle disorders
J96.00	Respiratory failure	K59.03	Functional intestinal disorders	M62.069	Muscle disorders
J96.01	Respiratory failure	K59.04	Functional intestinal disorders	M62.07	Muscle disorders
J96.02	Respiratory failure	K59.09	Functional intestinal disorders	M62.071	Muscle disorders
J96.1	Respiratory failure	K59.1	Functional intestinal disorders	M62.072	Muscle disorders

J96.10	Respiratory failure	K59.2	Functional intestinal disorders	M62.079	Muscle disorders
J96.11	Respiratory failure	K59.3	Functional intestinal disorders	M62.08	Muscle disorders
J96.12	Respiratory failure	K59.31	Functional intestinal disorders	M62.1	Muscle disorders
J96.2	Respiratory failure	K59.39	Functional intestinal disorders	M62.10	Muscle disorders
J96.20	Respiratory failure	K59.4	Functional intestinal disorders	M62.11	Muscle disorders
J96.21	Respiratory failure	K59.8	Functional intestinal disorders	M62.111	Muscle disorders
J96.22	Respiratory failure	K59.81	Functional intestinal disorders	M62.112	Muscle disorders
J96.9	Respiratory failure	K59.89	Functional intestinal disorders	M62.119	Muscle disorders
J96.90	Respiratory failure	K59.9	Functional intestinal disorders	M62.12	Muscle disorders
J96.91	Respiratory failure	N18	Chronic kidney disease (CKD)	M62.121	Muscle disorders
J96.92	Respiratory failure	N18.1	Chronic kidney disease (CKD)	M62.122	Muscle disorders
J98	Other respiratory disorders	N18.2	Chronic kidney disease (CKD)	M62.129	Muscle disorders
J98.0	Other respiratory disorders	N18.3	Chronic kidney disease (CKD)	M62.13	Muscle disorders
J98.01	Other respiratory disorders	N18.30	Chronic kidney disease (CKD)	M62.131	Muscle disorders
J98.09	Other respiratory disorders	N18.31	Chronic kidney disease (CKD)	M62.132	Muscle disorders
J98.1	Other respiratory disorders	N18.32	Chronic kidney disease (CKD)	M62.139	Muscle disorders
J98.11	Other respiratory disorders	N18.4	Chronic kidney disease (CKD)	M62.14	Muscle disorders
J98.19	Other respiratory disorders	N18.5	Chronic kidney disease (CKD)	M62.141	Muscle disorders
J98.2	Other respiratory disorders	N18.6	Chronic kidney disease (CKD)	M62.142	Muscle disorders
J98.3	Other respiratory disorders	N18.9	Chronic kidney disease (CKD)	M62.149	Muscle disorders
J98.4	Other respiratory disorders	M25	Joint disorder	M62.15	Muscle disorders
J98.5	Other respiratory disorders	M25.0	Joint disorder	M62.151	Muscle disorders
J98.51	Other respiratory disorders	M25.00	Joint disorder	M62.152	Muscle disorders

J98.59	Other respiratory disorders	M25.01	Joint disorder	M62.159	Muscle disorders
J98.6	Other respiratory disorders	M25.011	Joint disorder	M62.16	Muscle disorders
J98.8	Other respiratory disorders	M25.012	Joint disorder	M62.161	Muscle disorders
J98.9	Other respiratory disorders	M25.019	Joint disorder	M62.162	Muscle disorders
Z99.0	Dependence on Respirator or Oxygen	M25.02	Joint disorder	M62.169	Muscle disorders
Z99.1	Dependence on Respirator or Oxygen	M25.021	Joint disorder	M62.17	Muscle disorders
Z99.11	Dependence on Respirator or Oxygen	M25.022	Joint disorder	M62.171	Muscle disorders
Z99.12	Dependence on Respirator or Oxygen	M25.029	Joint disorder	M62.172	Muscle disorders
Z99.81	Dependence on Respirator or Oxygen	M25.03	Joint disorder	M62.179	Muscle disorders
R26	Abnormalities of gait and mobility	M25.031	Joint disorder	M62.18	Muscle disorders
R26.0	Abnormalities of gait and mobility	M25.032	Joint disorder	M62.2	Muscle disorders
R26.1	Abnormalities of gait and mobility	M25.039	Joint disorder	M62.20	Muscle disorders
R26.2	Abnormalities of gait and mobility	M25.04	Joint disorder	M62.21	Muscle disorders
R26.8	Abnormalities of gait and mobility	M25.041	Joint disorder	M62.211	Muscle disorders
R26.81	Abnormalities of gait and mobility	M25.042	Joint disorder	M62.212	Muscle disorders
R26.89	Abnormalities of gait and mobility	M25.049	Joint disorder	M62.219	Muscle disorders
R26.9	Abnormalities of gait and mobility	M25.05	Joint disorder	M62.22	Muscle disorders
R43	Cognition, perception, or emotional state symptoms	M25.051	Joint disorder	M62.221	Muscle disorders
R43.0	Cognition, perception, or emotional state symptoms	M25.052	Joint disorder	M62.222	Muscle disorders
R43.1	Cognition, perception, or emotional state symptoms	M25.059	Joint disorder	M62.229	Muscle disorders
R43.2	Cognition, perception, or emotional state symptoms	M25.06	Joint disorder	M62.23	Muscle disorders
R43.8	Cognition, perception, or emotional state symptoms	M25.061	Joint disorder	M62.231	Muscle disorders
R43.9	Cognition, perception, or emotional state symptoms	M25.062	Joint disorder	M62.232	Muscle disorders

R42	Cognition, perception, or emotional state symptoms	M25.069	Joint disorder	M62.239	Muscle disorders
R51	Headache	M25.07	Joint disorder	M62.24	Muscle disorders
R51.0	Headache	M25.071	Joint disorder	M62.241	Muscle disorders
R51.9	Headache	M25.072	Joint disorder	M62.242	Muscle disorders
R53	Malaise and fatigue	M25.073	Joint disorder	M62.249	Muscle disorders
R53.0	Malaise and fatigue	M25.074	Joint disorder	M62.25	Muscle disorders
R53.1	Malaise and fatigue	M25.075	Joint disorder	M62.251	Muscle disorders
R53.2	Malaise and fatigue	M25.076	Joint disorder	M62.252	Muscle disorders
R53.8	Malaise and fatigue	M25.08	Joint disorder	M62.259	Muscle disorders
R53.81	Malaise and fatigue	M25.1	Joint disorder	M62.26	Muscle disorders
R53.82	Malaise and fatigue	M25.10	Joint disorder	M62.261	Muscle disorders
R53.83	Malaise and fatigue	M25.11	Joint disorder	M62.262	Muscle disorders
G44	Headache	M25.111	Joint disorder	M62.269	Muscle disorders
G44.0	Headache	M25.112	Joint disorder	M62.27	Muscle disorders
G44.00	Headache	M25.119	Joint disorder	M62.271	Muscle disorders
G44.001	Headache	M25.12	Joint disorder	M62.272	Muscle disorders
G44.009	Headache	M25.121	Joint disorder	M62.279	Muscle disorders
G44.01	Headache	M25.122	Joint disorder	M62.28	Muscle disorders
G44.011	Headache	M25.129	Joint disorder	M62.3	Muscle disorders
G44.019	Headache	M25.13	Joint disorder	M62.4	Muscle disorders
G44.02	Headache	M25.131	Joint disorder	M62.40	Muscle disorders
G44.021	Headache	M25.132	Joint disorder	M62.41	Muscle disorders
G44.029	Headache	M25.139	Joint disorder	M62.411	Muscle disorders

G44.03	Headache	M25.14	Joint disorder	M62.412	Muscle disorders
G44.031	Headache	M25.141	Joint disorder	M62.419	Muscle disorders
G44.039	Headache	M25.142	Joint disorder	M62.42	Muscle disorders
G44.04	Headache	M25.149	Joint disorder	M62.421	Muscle disorders
G44.041	Headache	M25.15	Joint disorder	M62.422	Muscle disorders
G44.049	Headache	M25.151	Joint disorder	M62.429	Muscle disorders
G44.05	Headache	M25.152	Joint disorder	M62.43	Muscle disorders
G44.051	Headache	M25.159	Joint disorder	M62.431	Muscle disorders
G44.059	Headache	M25.16	Joint disorder	M62.432	Muscle disorders
G44.09	Headache	M25.161	Joint disorder	M62.439	Muscle disorders
G44.091	Headache	M25.162	Joint disorder	M62.44	Muscle disorders
G44.099	Headache	M25.169	Joint disorder	M62.441	Muscle disorders
G44.1	Headache	M25.17	Joint disorder	M62.442	Muscle disorders
G44.2	Headache	M25.171	Joint disorder	M62.449	Muscle disorders
G44.20	Headache	M25.172	Joint disorder	M62.45	Muscle disorders
G44.201	Headache	M25.173	Joint disorder	M62.451	Muscle disorders
G44.209	Headache	M25.174	Joint disorder	M62.452	Muscle disorders
G44.21	Headache	M25.175	Joint disorder	M62.459	Muscle disorders
G44.211	Headache	M25.176	Joint disorder	M62.46	Muscle disorders
G44.219	Headache	M25.18	Joint disorder	M62.461	Muscle disorders
G44.22	Headache	M25.2	Joint disorder	M62.462	Muscle disorders
G44.221	Headache	M25.20	Joint disorder	M62.469	Muscle disorders
G44.229	Headache	M25.21	Joint disorder	M62.47	Muscle disorders

G44.3	Headache	M25.211	Joint disorder	M62.471	Muscle disorders
G44.30	Headache	M25.212	Joint disorder	M62.472	Muscle disorders
G44.301	Headache	M25.219	Joint disorder	M62.479	Muscle disorders
G44.309	Headache	M25.22	Joint disorder	M62.48	Muscle disorders
G44.31	Headache	M25.221	Joint disorder	M62.49	Muscle disorders
G44.311	Headache	M25.222	Joint disorder	M62.5	Muscle disorders
G44.319	Headache	M25.229	Joint disorder	M62.50	Muscle disorders
G44.32	Headache	M25.23	Joint disorder	M62.51	Muscle disorders
G44.321	Headache	M25.231	Joint disorder	M62.511	Muscle disorders
G44.329	Headache	M25.232	Joint disorder	M62.512	Muscle disorders
G44.4	Headache	M25.239	Joint disorder	M62.519	Muscle disorders
G44.40	Headache	M25.24	Joint disorder	M62.52	Muscle disorders
G44.41	Headache	M25.241	Joint disorder	M62.521	Muscle disorders
G44.5	Headache	M25.242	Joint disorder	M62.522	Muscle disorders
G44.51	Headache	M25.249	Joint disorder	M62.529	Muscle disorders
G44.52	Headache	M25.25	Joint disorder	M62.53	Muscle disorders
G44.53	Headache	M25.251	Joint disorder	M62.531	Muscle disorders
G44.59	Headache	M25.252	Joint disorder	M62.532	Muscle disorders
G44.8	Headache	M25.259	Joint disorder	M62.539	Muscle disorders
G44.81	Headache	M25.26	Joint disorder	M62.54	Muscle disorders
G44.82	Headache	M25.261	Joint disorder	M62.541	Muscle disorders
G44.83	Headache	M25.262	Joint disorder	M62.542	Muscle disorders
G44.84	Headache	M25.269	Joint disorder	M62.549	Muscle disorders

G44.85	Headache	M25.27	Joint disorder	M62.55	Muscle disorders
G44.86	Headache	M25.271	Joint disorder	M62.551	Muscle disorders
G44.89	Headache	M25.272	Joint disorder	M62.552	Muscle disorders
G47	Sleep disorders	M25.279	Joint disorder	M62.559	Muscle disorders
G47.0	Sleep disorders	M25.28	Joint disorder	M62.56	Muscle disorders
G47.00	Sleep disorders	M25.3	Joint disorder	M62.561	Muscle disorders
G47.01	Sleep disorders	M25.30	Joint disorder	M62.562	Muscle disorders
G47.09	Sleep disorders	M25.31	Joint disorder	M62.569	Muscle disorders
G47.1	Sleep disorders	M25.311	Joint disorder	M62.57	Muscle disorders
G47.10	Sleep disorders	M25.312	Joint disorder	M62.571	Muscle disorders
G47.11	Sleep disorders	M25.319	Joint disorder	M62.572	Muscle disorders
G47.12	Sleep disorders	M25.32	Joint disorder	M62.579	Muscle disorders
G47.13	Sleep disorders	M25.321	Joint disorder	M62.58	Muscle disorders
G47.14	Sleep disorders	M25.322	Joint disorder	M62.59	Muscle disorders
G47.19	Sleep disorders	M25.329	Joint disorder	M62.5A	Muscle disorders
G47.2	Sleep disorders	M25.33	Joint disorder	M62.5A0	Muscle disorders
G47.20	Sleep disorders	M25.331	Joint disorder	M62.5A1	Muscle disorders
G47.21	Sleep disorders	M25.332	Joint disorder	M62.5A2	Muscle disorders
G47.22	Sleep disorders	M25.339	Joint disorder	M62.5A9	Muscle disorders
G47.23	Sleep disorders	M25.34	Joint disorder	M62.8	Muscle disorders
G47.24	Sleep disorders	M25.341	Joint disorder	M62.81	Muscle disorders
G47.25	Sleep disorders	M25.342	Joint disorder	M62.82	Muscle disorders
G47.26	Sleep disorders	M25.349	Joint disorder	M62.83	Muscle disorders

G47.27	Sleep disorders	M25.35	Joint disorder	M62.830	Muscle disorders
G47.29	Sleep disorders	M25.351	Joint disorder	M62.831	Muscle disorders
G47.3	Sleep disorders	M25.352	Joint disorder	M62.838	Muscle disorders
G47.30	Sleep disorders	M25.359	Joint disorder	M62.84	Muscle disorders
G47.31	Sleep disorders	M25.36	Joint disorder	M62.89	Muscle disorders
G47.32	Sleep disorders	M25.361	Joint disorder	M62.9	Muscle disorders
G47.33	Sleep disorders	M25.362	Joint disorder	M25.42	Joint disorder
G47.34	Sleep disorders	M25.369	Joint disorder	M25.421	Joint disorder
G47.35	Sleep disorders	M25.37	Joint disorder	M25.422	Joint disorder
G47.36	Sleep disorders	M25.371	Joint disorder	M25.429	Joint disorder
G47.37	Sleep disorders	M25.372	Joint disorder	M25.43	Joint disorder
G47.39	Sleep disorders	M25.373	Joint disorder	M25.431	Joint disorder
G47.4	Sleep disorders	M25.374	Joint disorder	M25.432	Joint disorder
G47.41	Sleep disorders	M25.375	Joint disorder	M25.439	Joint disorder
G47.411	Sleep disorders	M25.376	Joint disorder	G47.52	Sleep disorders
G47.419	Sleep disorders	M25.39	Joint disorder	G47.53	Sleep disorders
G47.42	Sleep disorders	M25.4	Joint disorder	G47.54	Sleep disorders
G47.421	Sleep disorders	M25.40	Joint disorder	G47.59	Sleep disorders
G47.429	Sleep disorders	M25.41	Joint disorder	G47.6	Sleep disorders
G47.5	Sleep disorders	M25.411	Joint disorder	G47.61	Sleep disorders
G47.50	Sleep disorders	M25.412	Joint disorder	G47.62	Sleep disorders
G47.51	Sleep disorders	M25.419	Joint disorder	G47.63	Sleep disorders
