# Supplementary material for: Comparison of long COVID, recovered COVID, and non-COVID Post-Acute Infection Syndromes over three years
Source: PLoS One. 2025 May 20;20(5):e0323104. doi: 10.1371/journal.pone.0323104 (PMC12092011; doi:10.1371/journal.pone.0323104)
Supplement: Attachment — Submitted filename: Response to Reviewers_PLOS_One_Final.docx [file pone.0323104.s005.docx]

PONE-D-24-52642R1

**Comparison of long COVID, recovered COVID, and non-COVID Post-Acute Infection Syndromes over three years**

PLOS ONE

Journal Requirements:

Please review your reference list to ensure that it is complete and correct. If you have cited papers that have been retracted, please include the rationale for doing so in the manuscript text, or remove these references and replace them with relevant current references. Any changes to the reference list should be mentioned in the rebuttal letter that accompanies your revised manuscript. If you need to cite a retracted article, indicate the article’s retracted status in the References list and also include a citation and full reference for the retraction notice.

The reference list has been checked for completion and correctness. The only change was the addition of three references in relation to point #6 below.

Additional Editor Comments:

This is an excellent paper. Finally, please see my comments.

#1. Long COVID -> long COVID

This change has been implemented throughout the text and figures.

#2. Recovered COVID patients -> "r"ecovered patients with COVID-19

- Please use the patient-first language.

This change has been implemented throughout the text and figures.

#3. Non-COVID -> "n"on-COVID

This change has been implemented throughout the text and figures.

#4. Charlson Comorbidity Index -> Charlson comorbidity index

This change has been implemented throughout the text and figures.

#5. In data section, the authors describe " smoking exposure (current, former, none", but in Table 1, yes or no.

Current and former smokers were grouped as yes for smoking exposure, while “none” smokers were marked as no. The text in the data section has been modified to clearly state this classification

#6. Likewise, many of the organ systems affected by the symptoms and diagnoses identified in the period >30 days following the index SARS-CoV-2 test, such as cardiovascular [#1], neurologic [#2], and musculoskeletal [#3], have been well documented to be associated with Long COVID [3, 32, 33]

Please add the top-tier paper reference. [1-3]

The text and references have been modified to include references specifically for cardiovascular, neurologic, and musculoskeletal related issues, which are referenced in the manner specified above.
